# VEuPathDB: the eukaryotic pathogen, vector and host bioinformatics resource center in 2023

**DOI:** 10.1093/nar/gkad1003

**Published:** 2023-11-11

**Authors:** Jorge Alvarez-Jarreta, Beatrice Amos, Cristina Aurrecoechea, Saikou Bah, Matthieu Barba, Ana Barreto, Evelina Y Basenko, Robert Belnap, Ann Blevins, Ulrike Böhme, John Brestelli, Stuart Brown, Danielle Callan, Lahcen I Campbell, George K Christophides, Kathryn Crouch, Helen R Davison, Jeremy D DeBarry, Richard Demko, Ryan Doherty, Yikun Duan, Walter Dundore, Sarah Dyer, Dave Falke, Steve Fischer, Bindu Gajria, Daniel Galdi, Gloria I Giraldo-Calderón, Omar S Harb, Elizabeth Harper, Danica Helb, Connor Howington, Sufen Hu, Jay Humphrey, John Iodice, Andrew Jones, John Judkins, Sarah A Kelly, Jessica C Kissinger, Nupur Kittur, Dae Kun Kwon, Kristopher Lamoureux, Wei Li, Disha Lodha, Robert M MacCallum, Gareth Maslen, Mary Ann McDowell, Jeremy Myers, Mustafa Veysi Nural, David S Roos, Samuel S C Rund, Achchuthan Shanmugasundram, Vasily Sitnik, Drew Spruill, David Starns, Sheena Shah Tomko, Haiming Wang, Susanne Warrenfeltz, Robert Wieck, Paul A Wilkinson, Jie Zheng

**Affiliations:** European Bioinformatics Institute, Hinxton CB10 1SD, UK; Institute of Systems, Molecular and Integrative Biology, University of Liverpool, Liverpool L69 7ZB, UK; University of Georgia, Athens, GA 30602, USA; School of Infection and Immunity, University of Glasgow, Glasgow, UK; European Bioinformatics Institute, Hinxton CB10 1SD, UK; University of Pennsylvania, Philadelphia, PA 19104, USA; Institute of Systems, Molecular and Integrative Biology, University of Liverpool, Liverpool L69 7ZB, UK; University of Georgia, Athens, GA 30602, USA; University of Pennsylvania School of Veterinary Medicine, Philadelphia, PA 19104, USA; University of Georgia, Athens, GA 30602, USA; University of Pennsylvania, Philadelphia, PA 19104, USA; University of Pennsylvania, Philadelphia, PA 19104, USA; University of Pennsylvania, Philadelphia, PA 19104, USA; European Bioinformatics Institute, Hinxton CB10 1SD, UK; Imperial College London, South Kensington, London SW7 2BU, UK; School of Infection and Immunity, University of Glasgow, Glasgow, UK; Institute of Systems, Molecular and Integrative Biology, University of Liverpool, Liverpool L69 7ZB, UK; University of Georgia, Athens, GA 30602, USA; University of Pennsylvania, Philadelphia, PA 19104, USA; University of Pennsylvania, Philadelphia, PA 19104, USA; University of Pennsylvania, Philadelphia, PA 19104, USA; University of Georgia, Athens, GA 30602, USA; European Bioinformatics Institute, Hinxton CB10 1SD, UK; University of Georgia, Athens, GA 30602, USA; University of Pennsylvania, Philadelphia, PA 19104, USA; University of Pennsylvania, Philadelphia, PA 19104, USA; University of Pennsylvania, Philadelphia, PA 19104, USA; University of Notre Dame, Notre Dame, IN 46556, USA; University of Pennsylvania, Philadelphia, PA 19104, USA; University of Pennsylvania, Philadelphia, PA 19104, USA; University of Pennsylvania, Philadelphia, PA 19104, USA; University of Notre Dame, Notre Dame, IN 46556, USA; University of Pennsylvania, Philadelphia, PA 19104, USA; University of Georgia, Athens, GA 30602, USA; University of Pennsylvania, Philadelphia, PA 19104, USA; Institute of Systems, Molecular and Integrative Biology, University of Liverpool, Liverpool L69 7ZB, UK; University of Pennsylvania, Philadelphia, PA 19104, USA; Imperial College London, South Kensington, London SW7 2BU, UK; University of Georgia, Athens, GA 30602, USA; University of Georgia, Athens, GA 30602, USA; University of Notre Dame, Notre Dame, IN 46556, USA; University of Georgia, Athens, GA 30602, USA; University of Pennsylvania, Philadelphia, PA 19104, USA; European Bioinformatics Institute, Hinxton CB10 1SD, UK; Imperial College London, South Kensington, London SW7 2BU, UK; Imperial College London, South Kensington, London SW7 2BU, UK; University of Notre Dame, Notre Dame, IN 46556, USA; University of Pennsylvania, Philadelphia, PA 19104, USA; University of Georgia, Athens, GA 30602, USA; University of Pennsylvania, Philadelphia, PA 19104, USA; University of Notre Dame, Notre Dame, IN 46556, USA; Institute of Systems, Molecular and Integrative Biology, University of Liverpool, Liverpool L69 7ZB, UK; Genomics England Limited, London E14 5AB, UK; European Bioinformatics Institute, Hinxton CB10 1SD, UK; University of Georgia, Athens, GA 30602, USA; Institute of Systems, Molecular and Integrative Biology, University of Liverpool, Liverpool L69 7ZB, UK; University of Pennsylvania, Philadelphia, PA 19104, USA; University of Georgia, Athens, GA 30602, USA; University of Georgia, Athens, GA 30602, USA; University of Notre Dame, Notre Dame, IN 46556, USA; Institute of Systems, Molecular and Integrative Biology, University of Liverpool, Liverpool L69 7ZB, UK; University of Pennsylvania, Philadelphia, PA 19104, USA

## Abstract

The Eukaryotic Pathogen, Vector and Host Informatics Resource (VEuPathDB, https://veupathdb.org) is a Bioinformatics Resource Center funded by the National Institutes of Health with additional funding from the Wellcome Trust. VEuPathDB supports >600 organisms that comprise invertebrate vectors, eukaryotic pathogens (protists and fungi) and relevant free-living or non-pathogenic species or hosts. Since 2004, VEuPathDB has analyzed omics data from the public domain using contemporary bioinformatic workflows, including orthology predictions via OrthoMCL, and integrated the analysis results with analysis tools, visualizations, and advanced search capabilities. The unique data mining platform coupled with >3000 pre-analyzed data sets facilitates the exploration of pertinent omics data in support of hypothesis driven research. Comparisons are easily made across data sets, data types and organisms. A Galaxy workspace offers the opportunity for the analysis of private large-scale datasets and for porting to VEuPathDB for comparisons with integrated data. The MapVEu tool provides a platform for exploration of spatially resolved data such as vector surveillance and insecticide resistance monitoring. To address the growing body of omics data and advances in laboratory techniques, VEuPathDB has added several new data types, searches and features, improved the Galaxy workspace environment, redesigned the MapVEu interface and updated the infrastructure to accommodate these changes.

## Graphical Abstract

**Graphical Abstract:**
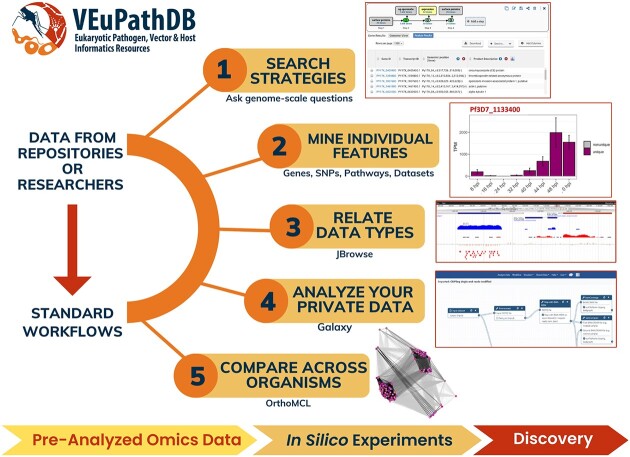

## Introduction

The Eukaryotic Pathogen, Vector and Host Informatics Resource (VEuPathDB, https://veupathdb.org) provides centralized access to omics data and computational tools and promotes data sharing among research communities that study invertebrate vectors, eukaryotic pathogens (protists and fungi) and relevant free-living or non-pathogenic species or hosts. First funded in 2004 by the NIAID (https://www.niaid.nih.gov/) as a Bioinformatics Resource Center (https://www.niaid.nih.gov/research/bioinformatics-resource-centers), VEuPathDB now has additional funding from Wellcome Trust (https://wellcome.org/), and has grown to support over 600 organisms with a wide range of pre-analyzed omics data, advanced search capabilities and data visualizations in an accessible web environment. VEuPathDB’s 14 projects (Table 1) share the same web architecture, tools and features, but differ in the organisms supported and underlying data (Table 1). Parasite, vector and host data exploration are supported by 13 projects, while OrthoMCL DB provides a platform for investigating orthology relationships across VEuPathDB organisms as well as species from archaea, bacteria, and eukaryotes that are not supported in VEuPathDB.

**Table 1.** VEuPathDB resources and organisms supported

Project	Web address	URL to access list of organisms supported	Number of datasets (release 65)
VEuPathDB	https://veupathdb.org	https://veupathdb.org/veupathdb/app/search/organism/GenomeDataTypes/result	3036
AmoebaDB	https://amoebadb.org	https://amoebadb.org/amoeba/app/search/organism/GenomeDataTypes/result	93
CryptoDB	https://cryptodb.org	https://cryptodb.org/cryptodb/app/search/organism/GenomeDataTypes/result	90
FungiDB	https://fungidb.org	https://fungidb.org/fungidb/app/search/organism/GenomeDataTypes/result	663
GiardiaDB	https://giardiadb.org	https://giardiadb.org/giardiadb/app/search/organism/GenomeDataTypes/result	65
HostDB	https://hostdb.org	https://hostdb.org/hostdb/app/search/organism/GenomeDataTypes/result	77
MicrosporidiaDB	https://microsporidiadb.org	https://microsporidiadb.org/micro/app/search/organism/GenomeDataTypes/result	75
PiroplasmaDB	https://piroplasmadb.org	https://piroplasmadb.org/piro/app/search/organism/GenomeDataTypes/result	55
PlasmoDB	https://plasmodb.org	https://plasmodb.org/plasmo/app/search/organism/GenomeDataTypes/result	308
ToxoDB	https://toxodb.org	https://toxodb.org/toxo/app/search/organism/GenomeDataTypes/result	171
TrichDB	https://trichdb.org	https://trichdb.org/trichdb/app/search/organism/GenomeDataTypes/result	33
TriTrypDB	https://tritrypdb.org	https://tritrypdb.org/tritrypdb/app/search/organism/GenomeDataTypes/result	274
VectorBase	https://vectorbase.org	https://vectorbase.org/vectorbase/app/search/organism/GenomeDataTypes/result	1286
OrthoMCL DB	https://orthomcl.org	https://orthomcl.org/orthomcl/app/release-summary	832 genomes represented^a)^

^a^Includes core and peripheral organisms.

VEuPathDB uses contemporary bioinformatic workflows to analyze and integrate data from public domains such as Sequence Read Archive (1) (https://www.ncbi.nlm.nih.gov/sra), InterPro (2) and GO Consortium (3,4). Data types include genome sequence and annotation, transcriptomics, proteomics, epigenomics, metabolomics, population resequencing, clinical data, surveillance data, host-pathogen interactions and orthology profiles across all integrated organisms. The results of these analyses form the cornerstone of the unique data mining platform comprising: Search Strategies for genome wide queries as part of *in silico* experiments, a records system that compiles all data for individual features (e.g. genes, SNPs, or metabolic pathways) and dynamic visualizations in a Genome Browser. Comparisons are easily made across data types, data sets and organisms in this uniquely flexible and tractable system.

The discovery of meaningful biological relationships that exist at the intersection of disparate data types is facilitated by VEuPathDB’s reference genome concept. Data mining strategies within VEuPathDB support the major goal of providing tools to make comparisons across the >600 organisms and >3000 functional data sets. To this end, in consultation with the research community, VEuPathDB designates one annotated genome as the ‘reference’ where multiple strains of an organism are integrated. All functional data are aligned to the chosen reference for comparisons across data types, while the all-organism orthology profiles relate the data across strains. The result is an expansive network of pre-analyzed data for closely as well as distantly related organisms in a powerful data mining platform designed to support hypothesis driven research.

The analysis of private data via Galaxy (5), a web-based platform for bioinformatic analyses, coupled with export tools developed in-house allow the exploration of private data in the context of public data already integrated into VEuPathDB. A specific advantage of the Galaxy platform is that workflows, series of analyses linking the output of one analysis to the input of a subsequent analysis, are created in a menu-driven, drag and drop interface, alleviating the need for command line programming. Workflows created by VEuPathDB are published in the VEuPathDB Galaxy server (https://veupathdbprod.globusgenomics.org/) Shared Data menu and linked to the home page. VEuPathDB workflows follow the methods used for the analysis of public data hosted on our sites, and preloaded reference genomes assist novice analysts with analyzing their own large-scale data sets. Private analysis results are easily ported to VEuPathDB where Search Strategies offer over 100 pre-configured queries based on integrated data that can be used to subset and explore the biological properties of the user's data.

MapVEu is the VEuPathDB tool for visualization, filtering, download and exploration of spatially resolved data such as vector surveillance data. The tool integrates genomic, phenotypic and population data for traits such as insecticide resistance, microsatellite variation, chromosomal inversions and abundance.

Recent memberships and accolades reinforce VEuPathDB’s value to the research community. The Global Biodata Coalition (https://globalbiodata.org/) recognizes VEuPathDB as a Global Core Biodata Resource (https://globalbiodata.org/what-we-do/global-core-biodata-resources/) whose long-term funding and sustainability are critical to life science and biomedical research worldwide. VEuPathDB is also a founding member of the NIAID Data Ecosystem (https://data.niaid.nih.gov), which facilitates the discovery of Infectious and Immune-mediated Disease (IID) data across many repositories. Metadata associated with VEuPathDB datasets is indexed and searchable on the NIAID Data Ecosystem Data Portal which is accessible worldwide. In addition, the DataWorks! Prize program is an annual challenge launched by The Federation of American Societies for Experimental Biology and the National Institutes of Health to showcase and reward bold and innovative practices supporting data sharing and reuse. Highlighting the reach and public value of this resource, VEuPathDB was a 2023 DataWorks! Prize Winner (https://datascience.nih.gov/director/directors-blog-dataworks-winners-2023) in two categories: Significant Achievement Award for Data Reuse and the People's Choice Award.

VEuPathDB’s careful and integrated design advances the reuse of omics data and supports hypothesis driven research, especially for research scientists who do not have computer programming experience. The tools and features in VEuPathDB provide means for interrogating large volumes of data to find relationships between genes and other features. In the last two years, VEuPathDB has added support for emerging data types, improved interoperability with new and updated tools, and made significant infrastructure improvements that support our spatially resolved data tool, MapVEu.

## New in VEuPathDB

Emerging technologies and community input drive development of new features and tools in VEuPathDB resources. In the past two years we have added support for new data types, tools that support workspace improvements and interoperability, and made significant infrastructure changes that support scalability, transparency and feature development.

### Data

VEuPathDB supports a wide range of data types including genome sequences and annotation, transcriptomics, proteomics, epigenomics, metabolomics, population resequencing, clinical data, surveillance data and host-response data. Our bimonthly releases add new data in these categories. Described below are data types added in the last two years.

#### Protein Structure Predictions

High-quality protein structure predictions serve as a valuable tool for generating and supporting biological hypotheses. Therefore, VEuPathDB has incorporated protein structure predictions from AlphaFold (6,7), a powerful artificial intelligence tool for predicting 3D protein structures. AlphaFold DB (8) (https://alphafold.ebi.ac.uk/) is a public database that contains predictions for over 200 million proteins. Models generated by AlphaFold can be used to predict protein function based on sequence and/or protein folding similarity, validate hypotheses, and more. Within VEuPathDB, two new record page features in the Structure Analysis section are available for genes with UniProt IDs that match AlphaFold DB or that have good protein sequence similarity to UniProt entries represented in AlphaFoldDB. The AlphaFold section on gene record pages tabulates predicted structures and provides access to AlphaFold record pages for detailed exploration. Additionally, the AlphaFold Structure Prediction Visualization section offers a simplified version of the AlphaFold 3D Viewer for convenient and easy visual inspection of protein features.

#### Single-cell transcriptomics

**Figure 1.**
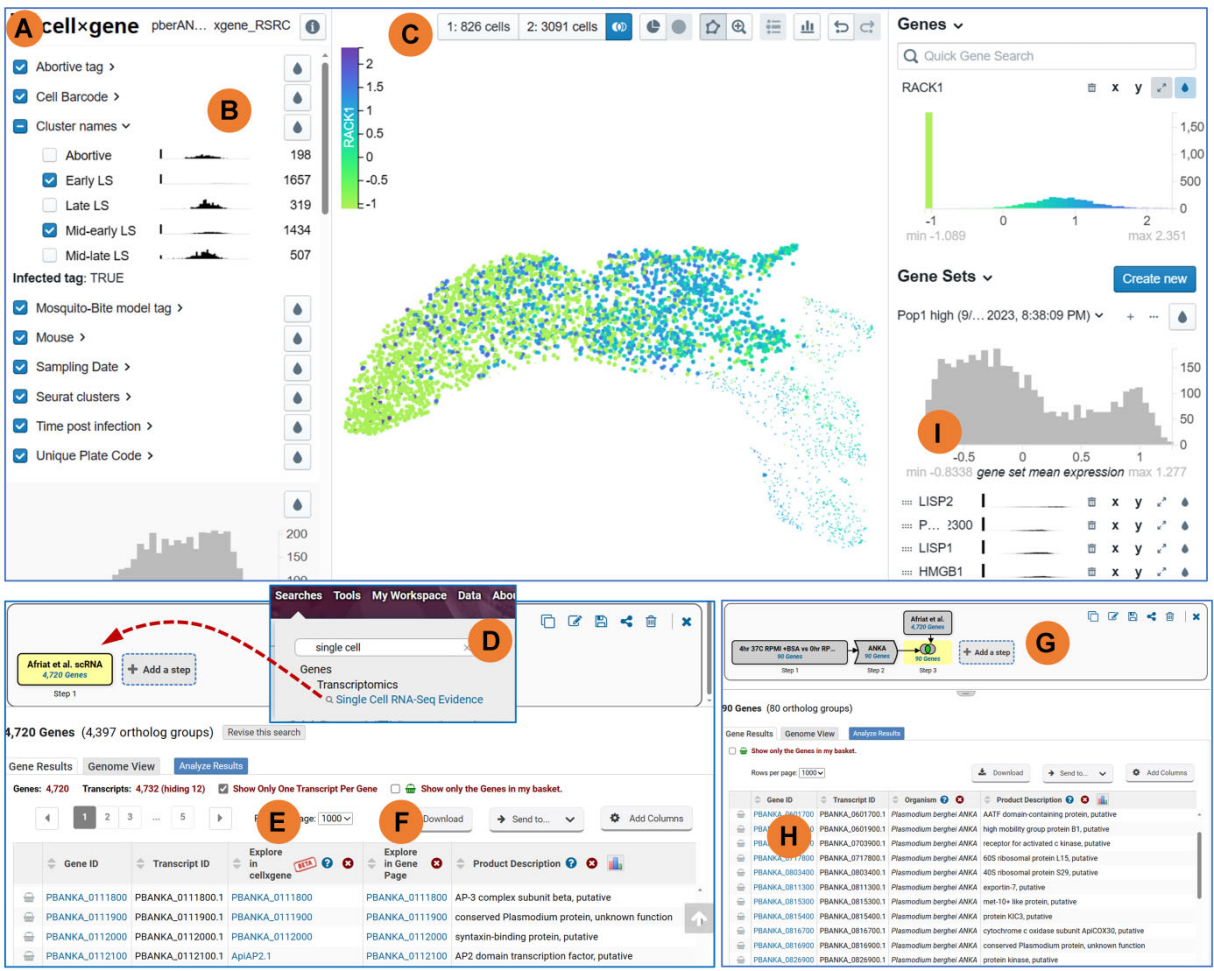
Single-cell Transcriptomics: a new data type supported in VEuPathDB. (**A**) CELLXGENE application for the visualization and analysis of scRNA-Seq data currently displaying data for RACK1, PBANAKA_0703900. (**B**) Interactive metadata display for exploring subsets based on experimental parameters. The cluster name category displays expression profiles for RACK1 in each sample. Check boxes are used for configuring the UMAP display and/or defining subgroups for differential expression analysis. (**C**) Tool set for subgroup selection and display. Center panel of the CELLXGENE app with interactive tools used for subgroup selection, initiating differential expression analysis or manipulating the UMAP cluster images displayed below the tool set. (**D**) The Genes by Single Cell RNA-Seq Evidence search returns all genes with data in a particular scRNA-Seq data set. Search results for the Genes by Single Cell RNA-Seq Evidence provide data columns for easy access to the CELLXGENE app (**E**) and the scRNA-Seq section of the gene's record page (**F**). (**G**) Multi-step strategy to investigate the correlation between bulk RNA-Seq in *Plasmodium vivax* with scRNA-Seq data in *Plasmodium berghei*. The full strategy can be found at https://plasmodb.org/plasmo/app/workspace/strategies/import/5f64cd2e2b16b8aa. (**H**) The first column of the search result table provides a link for easy access to gene pages. (**I**) The results of the differential expression configured in C are displayed in the Gene Sets panel and, in this case, reveal other known liver-specific genes.

Single-cell RNA sequence (scRNA-Seq) is a valuable new data type defining the transcriptomes of individual cells within a culture or tissue to reveal subpopulations that may offer novel treatment opportunities or insights into cellular biology. VEuPathDB supports scRNA-Seq data as UMAP (9) cell cluster projections and displays these data in the CELLXGENE (10) interactive data mining application (Figure 1A). For exploration of cell and tissue subpopulations, visualizations in CELLXGENE correlate cell UMAP projections with cell and experimental metadata (e.g. life cycle stage, infection route, time post infection) as well as expression measurements (Figure 1B). Differential expression between groups of cells chosen based on metadata or manual selection of the UMAP projection (Figure 1C) is easily accomplished in CELLXGENE and offers a powerful data mining and discovery tool. In addition to the fully-featured CELLxGENE application, a simple view of the UMAP projection is available on gene record pages, colored by the gene's expression profile.

#### Annotation improvements

Genome sequence and annotation provides the backbone for effective functional genomics inquiry within VEuPathDB. VEuPathDB supports annotation improvements through manual genome annotation of selected organisms from the literature, community annotation, and automated transfer of product descriptions for some genomes. All VEuPathDB reference genomes are now supported in Apollo, a community annotation platform where researchers can suggest edits to structural gene models and functional annotations such as gene names, product descriptions and GO terms. Periodically, VEuPathDB curators review community annotations and sync them with the VEuPathDB genome annotation. The Data Set Release History table on the record pages of genome sequence and annotation data sets chronicles additions from Apollo. To avoid multiple public representations of annotated genomes, VEuPathDB, with the permission of the data owners, works with data repositories to update the archival record with annotation improvements made at VEuPathDB. In addition, starting in September 2023, VEuPathDB electronically substitutes Pfam (11) domain descriptions for annotated gene product description in genomes with > 80% non-informative gene names such as ‘unspecified product’ or ‘hypothetical’. Electronically transferred gene product descriptions are amended with ‘domain containing protein’ and the details of the electronic transfer are chronicled in the gene page Product Descriptions table.

### Searches

The search strategy system encompasses over 100 preconfigured searches that query the VEuPathDB workflow results and return lists of features that meet the search criteria. To increase the data mining power of VEuPathDB and support the new data types, the following new searches have been developed.

#### Genes with AlphaFold predictions

**Figure 2.**
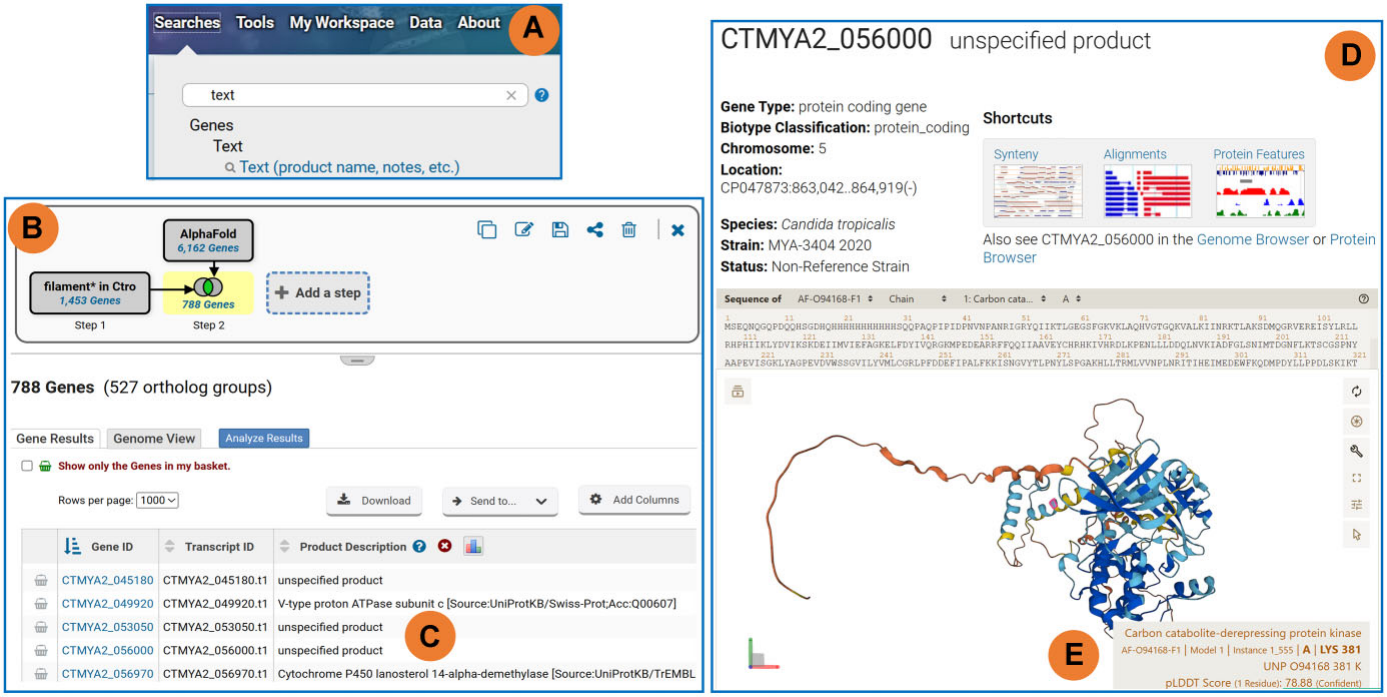
AlphaFold Structural Predictions: a new data type supported in VEuPathDB. (**A**) The Genes by Text search used to begin a two-step strategy to reveal genes involved in fungal filamentation. (**B**) Strategy graphic and result table. The Step 1 text search for ‘filament*’ includes the astrisk to broaden the query term to include plurals or compound words that begin with filament, e.g. filamentation, filaments. The text search results are intersected with the Genes by AlphaFold Predictions search to find 788 gens from the text search that have AlphaFold data. (**C**) Many genes returned by the two-step strategy lack specific gene product information and are classified as ‘unspecified product’. Gene page header (**D**) and AlphaFold data section (**E**) for the gene CTMYA2_05 600 which is classified as an unspecified product. (**E**) The AlphaFold structure prediction data support the identity of CTMYA2_05 600 as a possible Carbon catabolite derepressing protein kinase, which play a role in filamentation. The full strategy can be found at https://fungidb.org/fungidb/app/workspace/strategies/import/19cde8976530fcde.

The new Genes by AlphaFold Predictions search returns all genes with AlphaFold structural predictions for the organisms specified in the search criteria. Since protein structure can contribute to understanding a gene's function, AlphaFold structural prediction data can help elucidate or infer function on uncharacterized genes when used within a multi-step search strategy. For example, fungal pathogens such as *Candida tropicalis* can invade and colonize host organisms or establish biofilms in medical equipment (12), and filamentation plays a vital role in virulence. A two-step *in-silico* experiment to find *C. tropicalis* genes involved in fungal filamentation might begin with a text search for the term ‘filament*’ (Figure 2A) and the result can be further refined by intersecting a search for all genes with AlphaFold predictions (Figure 2B). Although genes returned by the strategy contain the term filament in their record, many are not well understood as indicated by product descriptions such as ‘unspecified product’ (Figure 2C). Further inspection of the AlphaFold visualizations for several genes in the strategy result reveals that the unspecified product, CTMYA2_056 000 (Figure 2D) may be a putative carbon catabolite-derepressing protein kinase (Figure 2E), which has an important role in fungal filamentation.

#### Genes with single-cell RNA-Seq data

As a point of entry to this new data type, a simple search returns all genes represented in a chosen scRNA-Seq experiment (Figure 1D). The search results page provides links to the interactive CELLxGENE application (Figure 1E) with experimental data preloaded, and to gene pages (Figure 1F) for every gene returned by the search.

While scRNA-Seq data defines the transcriptome of single cells, bulk RNA-Seq examines whole samples or tissues and can be used to confirm or corroborate scRNA-Seq data within VEuPathDB. The following example demonstrates the utility of VEUPathDB strategies integrated with the CELLXGENE analysis tools. Using microenvironments to mimic the *Plasmodium vivax* liver stages, Roth *et al.* (13) performed bulk RNA sequencing to reveal transcriptional changes. On the other hand, a recent scRNA-Seq study directly measured transcriptomes of individual cells in the early, mid-early, mid-late and late liver stages of *Plasmodium berghei* infection in mice (14). A multi-step strategy to compare these data (Figure 1G) begins with a search of the microenvironment data to return *Plasmodium vivax* genes that are likely expressed in liver-stage (Step 1). The *Plasmodium vivax* genes were transformed into their *Plasmodium berghei* orthologs (Step 2) for easy access to gene pages from the strategy result (Figure 1H). In step 3, the *Plasmodium berghei* orthologs are intersected with the single-cell liver stage data to confirm that scRNA-Seq data is available for all gene in the result (Figure 1G). Examining the CELLXGENE expression profile of PBANKA_0 703 900, RACK1, within each experimental sample (Figure 1B) corroborates the *Plasmodium vivax* bulk RNA-Seq data. Differential expression between the late/mid-late samples (826 cells) and the early/mid-early samples (3091 cells) (Figure 1C) reveals other known liver-specific genes (e.g. LISP1, LISP2) in the late/mid-late samples (Figure 1I).

#### Genes with unannotated intron junctions

The Unannotated Intron Junctions search (found under Gene Models in the Searches menu) enables users to identify genes that contain, or are flanked by, unannotated high confidence intron junction-spanning reads from RNA-seq data. These genes may be incompletely or inaccurately annotated due to missing introns/exons and/or alternative splice variants. Once genes with unannotated introns have been identified, users can explore them in JBrowse and correct gene structures in Apollo, an open-source software enabling users to inspect, refine and add gene models to the current genome annotations.

### General operations and workspaces

#### My organism preferences

**Figure 3.**
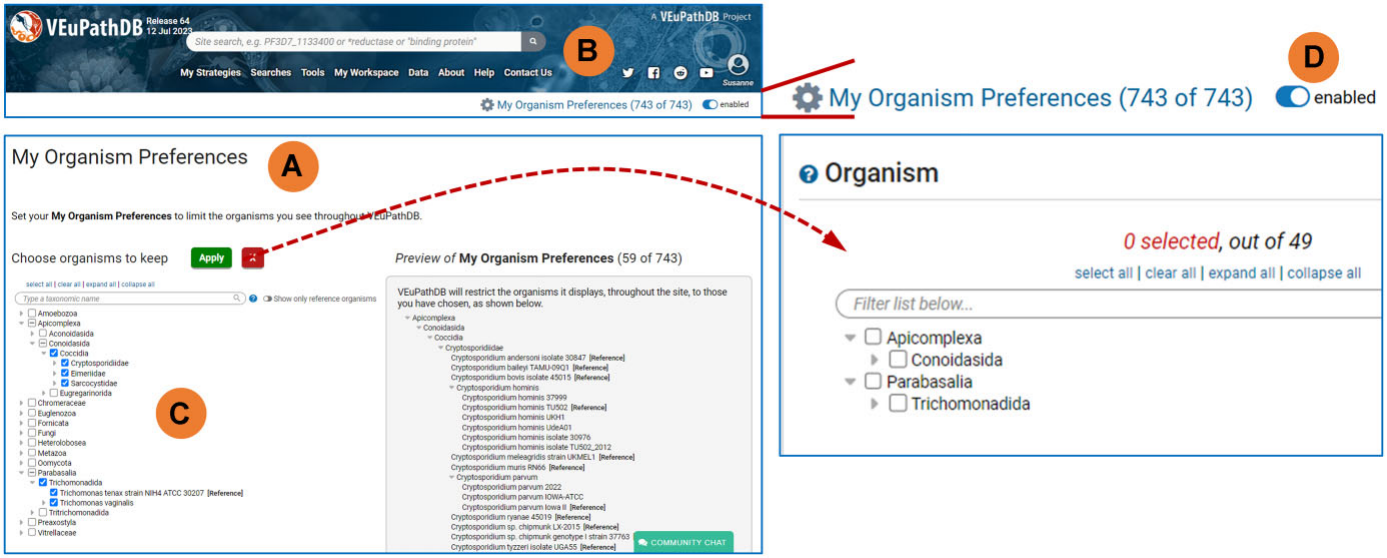
My Organism Preferences: a new tool for configuring menu displays. (**A**) The tool allows users to limit menu display to information pertaining to only their organisms of interest. Available from the header (**B**) of any page, the tool allows users to choose their preferred organisms from the tree of all organisms in the site. Choices made in the full tree are reflected on the right side of the tool. Once choices are applied (**C**), the site menus are limited to the user's organism preferences. (**D**) The tool is easily disabled with a toggle switch in the header.

VEuPathDB contains data on hundreds of organisms. This can result in an overwhelming number of hits in the site search and long menus in tools and searches. A new feature called ‘My Organism Preferences’ (Figure 3A) is accessed from the header (Figure 3B), and allows users to configure the menus to display data related to only selected organisms (Figure 3C). The tool allows broad or specific selections, with options to choose particular species or any other rank in the taxonomic hierarchy (Figure 3C). A simple toggle button (Figure 3D) lets users enable or disable this functionality to either apply the previously chosen preference or open the menus to all organisms in the database.

#### Download data files

VEuPathDB has implemented an improved tool for downloading genome scale files such as genome.fasta or GFF files for data loaded since 2015, with the exception of the tool implementation in VectorBase which contains files loaded since 2020. File folders are still available for accessing all download files, regardless of integration date. The new tool simplifies identification of files of interest, allowing users to filter by organism, VEuPathDB release, file content, data category and file format. Similar to other VEuPathDB tools, all data and files are available by default.

#### My data sets

The My Data Sets tool provides an interface for a user's private files and gene lists for further exploration in context with data already integrated into VEuPathDB. Tool access is provided in the My Workspace header under My data sets. Originally released as a hub for interacting with exports from VEuPathDB Galaxy, My Data Sets now accommodates uploads from your computer or a URL in the New Uploads tab, as well as imports from a search strategy via the Send To tool (see below). New uploads must be a txt file with Gene ID as the first column. Each uploaded file receives a record page that can be shared with other users. The All tab provides a table of file names that link to file record as well as other associated metadata about the file.

#### Send To tool

The results of searches that return genes can now easily be transferred to other tools or projects without the need for manually copying IDs into other tools or sites. The Send To tool assists with data management and tractability, saving lists or files for later use. The new tool copies the ID list from any gene search result to My Basket, a user's personal page for saving individual genes or features, or My Data Sets, a user's personal page for saving txt files, within the current project. Send To VEuPathDB is useful for interrogating orthology relationships across distantly related organisms. The tool uses the ID list as input for the Genes by ID search on VEuPathDB where all organisms are supported and the Transform by Orthology feature takes advantage of orthology profiles across all VEuPathDB organisms.

#### Galaxy workspace

To improve job and workflow performance, the Galaxy software was updated to version 20.9. In addition to the previous set of tools and workflows, we now offer additional tools, such as those for scRNA-seq (Scanpy, Seurat) (15,16), species identification (CryptoGenotyper) (17), table manipulation (Datamash) (https://www.gnu.org/software/datamash/) and proteomics (MSstats, Search GUI, Peptide Shaker, MaxQuant) (18–21). Available from the VEuPathDB Galaxy homepage is a new ChIP Seq workflow that approximates the VEuPathDB workflow and produces bigwig files that can be exported to VEuPathDB projects for inspection in the genome browser in context with other VEuPathDB integrated data.

#### Multi-sequence capable BLAST

The BLAST tool now supports multiple input sequences. The tool accommodates up to 100 sequences of up to 100 000 amino acids or 1 million nucleotides. Each submitted BLAST job is logged in the ‘My Jobs’ page and remains available indefinitely. Jobs that are run on multiple sequences include results in two formats. The Combined Result is a nonredundant list of all sequences with similarity to any input sequence. The Individual Result offers a separate result for each input sequence with the ability to toggle between result lists. Combined and individual results can be downloaded in multiple formats and the gene list of individual results can be exported to the strategy system for further data mining in VEuPathDB.

### System architecture and infrastructure

#### Exploratory data analysis infrastructure

VEuPathDB has developed an exploratory data analysis (EDA) platform that specializes in allowing users to examine datasets with complex structures in order to understand variables and the relationships between them. Subsets and slices of the data can be submitted to analysis algorithms and visualized using a suite of tools, with the goal of discovering patterns, finding outliers, and generating hypotheses for further testing. At this time the EDA drives the updated MapVEu tool, demonstrating that the EDA backend is scalable, having handled hierarchically structured datasets containing several million samples. We will expand the use of EDA in our websites as described in a later section.

#### MapVEu improvements

MapVEu is a powerful geoinformatics tool that displays scientific data in the context of an interactive global map and provides fast exploration of geospatial data, such as vector surveillance data. Opportunities to filter, zoom into locations and or plot variables can reveal relationships between the large number of variables that make up these complex structured data sets. Infrastructure and interface updates have significantly improved the tool.

**Figure 4.**
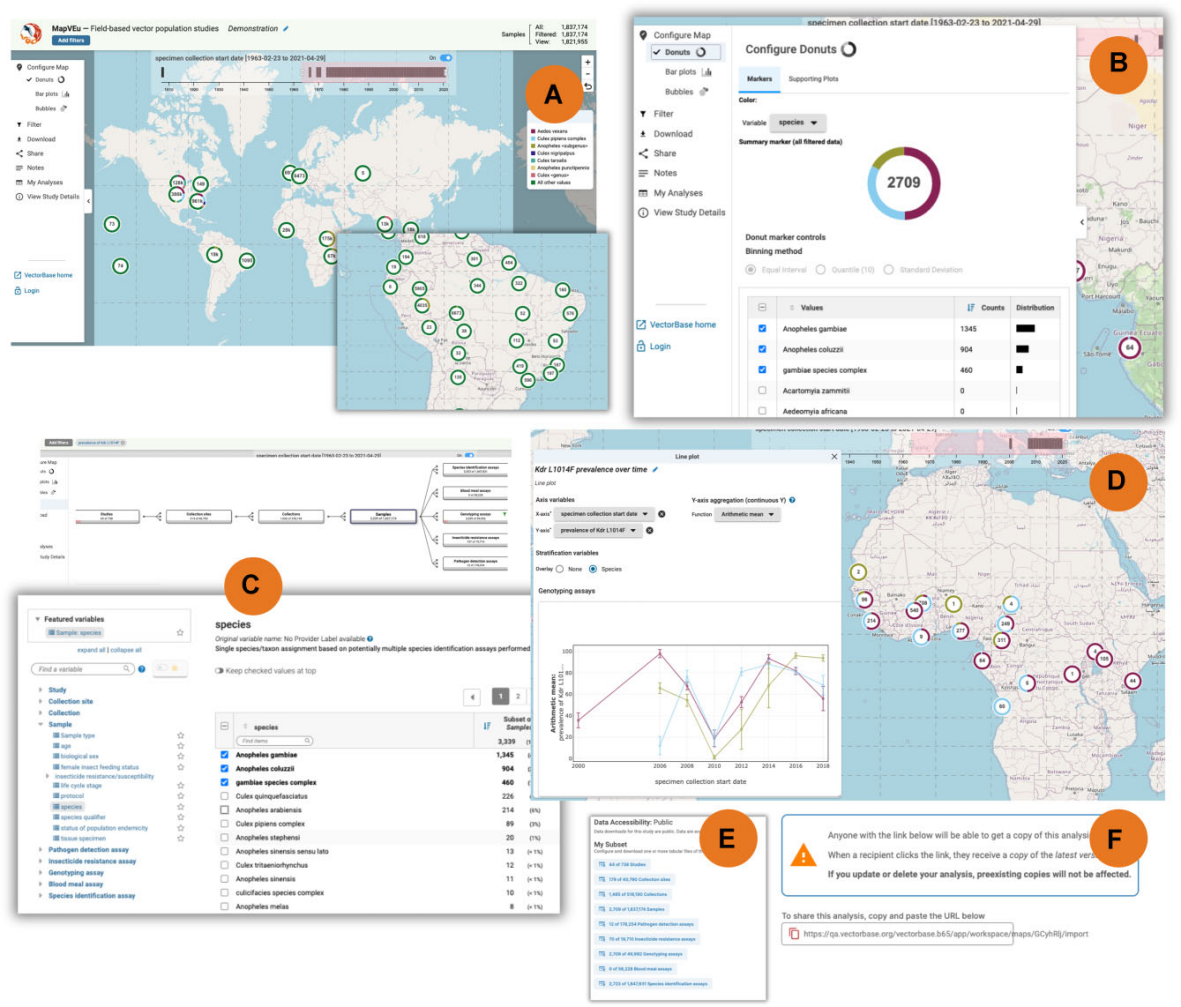
The MapVEu web application and exploratory data analysis (EDA) platform facilitates access to and exploration of geospatial data in VectorBase. Features of the MapVEu platform include a full-screen map with semantic zooming (**A**), wherein clicking on a marker causes it to zoom in and disaggregate. The user interface features a menu (**B**) that can be used to configure the map with markers to visualize any of the variables in the dataset (B), to filter continuous and categorical data points (**C**), and to make floating plots for data currently visible on the map (**D**). Shown here are donut markers for vector species, with the analysis restricted to records containing data for the prevalence of Kdr L1014F mutation associated with insecticide resistance, and a supporting plot visualizing trends in prevalence of Kdr 1014F mutation over a 20-year period for each of the selected species. All data can be downloaded as customizable flat files (**E**), and analyses can be shared (**F**).

Now underlying the tool is the new EDA infrastructure (details above) that expands MapVEu's data handling capabilities to easily manage data sets with millions of data points, presented in a full screen map with semantic zooming (Figure 4A). Data is structured hierarchically with each level having up to hundreds of metadata variables, both categorical and continuous. The location of data on the map is summarized with Markers that can be configured to represent any variable in one of three visualizations: donuts, bar plots or bubbles (Figure 4B). Filtering is accomplished with the VEuPathDB filter parameter, which features a searchable tree of data categories on the left that expands to reveal lists of variables. Once a variable is chosen, a details panel offers interactive tables and distribution data (Figure 4C).

Supporting plots, also a function of the EDA infrastructure, greatly expand the analysis capabilities with visualizations of any variable in the filtered data set, including X–Y relationships (scatter plot, line plot, time series), distributions (histogram, box plot), and counts and proportions (bar plot, contingency table) (Figure 4D). Supporting plots receive data from the map and, upon zoom or scroll of the map, will update to reflect data from that area. Users can download all data, or the filtered subset created (Figure 4E). Analyses are saved in the Supporting Plots tab as well as the My Analyses section. Notes can be added to the analyses and analyses can be shared using a stable link (Figure 4F).

## Outreach

The diverse research communities served by VEuPathDB reflect the breadth of organisms supported in the resource. The outreach team communicates directly with users and stakeholders to discuss data, receive feature suggestions, provide instruction, and ensure the resource quality. The VEuPathDB email hotline (help@veupathdb.org) and social media accounts enable direct communication with VEuPathDB staff. Video calls and virtual lab meetings are available upon request. Educational experiences include webinars, workshops, pdf training materials, and video tutorials. Webinars offered after each release (∼6 per year) provide an overview of new data and features, while special topics webinars focus on topics such as mining RNA-Seq data, gene record pages, the search strategy system, and community annotation. Webinar recordings are freely available (https://veupathdb.org/veupathdb/app/static-content/webinars.html). Each year VEuPathDB sponsors at least one in-person 4-day workshop in early summer, and one virtual multi-day workshop, usually in December, that emphasize hands-on training for each attendee with training materials publicly available from the workshop schedule (most recent virtual: https://veupathdb.org/veupathdb/app/static-content/workshopSept2022.html#Schedule). Additional workshops are offered in collaboration with sponsors such as Wellcome Connecting Science or associated with scientific conferences. The most recent educational materials for any subject are available on the Learning Materials page (https://veupathdb.org/veupathdb/app/static-content/tutorials.html). Video tutorials are available from our YouTube Channel (https://www.youtube.com/eupathdb).

## Future directions

VEuPathDB will continue to develop and integrate new tools and load new datasets to support community needs. We will continue to develop improved automation of data loading especially for those datasets that are available in established archival repositories and that are well described. In addition, critical datasets not available in repositories will be loaded directly from the community. To enhance our users' ability to explore and analyze data, we will expand the integration of the EDA system into other resource components such as MapVEu, the search strategy system and visualization components on record pages. Finally, the OrthoMCL website and algorithm will be revised, integrating orthology detection with OrthoFinder (22) and enhancing phylogenetic visualization tools.

## Data Availability

All data are available from the download tools on VEuPathDB project websites: AmoebaDB (https://amoebadb.org), CryptoDB (https://cryptodb.org), FungiDB (https://fungidb.org), GiardiaDB (https://giardiadb.org), MicrosporidiaDB (https://microsporidiadb.org), PiroplasmaDB (https://piroplasmadborg), PlasmoDB (https://plasmodb.org), ToxoDB (https://toxodb.org), TrichDB (https://trichdb.org), TriTrypDB (https://tritrypdb.org), VectorBase (https://vectorbase.org), and VEuPathDB (https://veupathdb.org). Project code can be found at our GitHub repository (https://github.com/VEuPathDB). Release 65 (September 12, 2023) of VEuPathDB contains over 3000 data sets. The release dates, versions and sources can be accessed at (https://veupathdb.org/veupathdb/app/search/dataset/AllDatasets/result) and links therein.
